# Differential diagnosis of clear cell renal cell carcinoma with low signal intensity on T2WI from angiomyolipoma without visible fat on MR imaging

**DOI:** 10.3389/fonc.2025.1564485

**Published:** 2025-03-28

**Authors:** Zi-xuan Chen, Yi Zhang, Shuai Ren, Ying-ying Cao, Qi Lan, Fan Xia, Zhong-qiu Wang, Wen-li Qiu

**Affiliations:** ^1^ Department of Radiology, Affiliated Hospital of Nanjing University of Chinese Medicine, Nanjing, China; ^2^ Department of Pathology, Affiliated Hospital of Nanjing University of Chinese Medicine, Nanjing, China

**Keywords:** AML.wovf, ccRCC, MRI, wedge-shaped sign, pseudocapsule formation, ADC

## Abstract

**Purpose:**

This study aimed to determine the potential of magnetic resonance imaging (MRI) parameters in differentiating between angiomyolipoma without visible fat (AML.wovf) and clear cell renal cell carcinoma (ccRCC) with low signal intensity on T2-weighted imaging (T2WI).

**Materials and methods:**

This is a retrospective study involving 36 cases of ccRCC and 17 cases of AML.wovf from September 2016 to July 2023. All patients underwent histological examination on resected specimens and contrast-enhanced magnetic resonance imaging (CE-MRI). Clinical characteristics such as age, gender, and symptoms of hematuria and lumbago were recorded. A panel of MRI parameters were analyzed, including the tumor growth patterns, the wedge-shaped sign, pseudocapsule formation, the arterial-to-delayed enhancement ratio (ADER), and the apparent diffusion coefficient (ADC). The potential of these MRI parameters in distinguishing ccRCC from AML.wovf was finally determined and visualized in a nomogram.

**Results:**

There were no significant differences in age, gender, and clinical symptoms between the ccRCC and AML.wovf groups. The wedge-shaped sign was more prevalent in patients with AML.wovf (*p* = 0.027), while pseudocapsule formation was mainly observed in cases of ccRCC (*p* < 0.001). Quantitative MRI revealed a significantly lower ADC in patients with AML.wovf (*p* = 0.007). Pseudocapsule formation (OR = 140.29, *p* = 0.004), the wedge-shaped sign (OR = 0.05, *p* = 0.047), and ADC (OR = 36.22, *p* = 0.037) were independent predictors for differentiating between AML.wovf and ccRCC, and their combination demonstrated the highest diagnostic accuracy, with an area under the curve (AUC) of 0.913 in the receiver operating characteristic (ROC) analysis.

**Conclusion:**

A combination of MRI parameters, including the wedge-shaped sign, pseudocapsule formation, and ADC, can accurately differentiate between AML.wovf and ccRCC.

## Introduction

Clear cell renal cell carcinoma (ccRCC) is the dominant subtype of renal cell carcinoma (RCC), representing approximately 80% of adult renal malignancies (1). Renal angiomyolipoma (AML) is the most prevalent subtype of benign renal tumors, typically showing visible fat on computed tomography (CT) or magnetic resonance imaging (MRI) (2). However, angiomyolipoma without visible fat (AML.wovf) is a benign mass in the kidney containing less than 10% of fat component, leading to challenges in imaging diagnosis (3, 4). Due to the lack of fat component, AML.wovf is homogeneously hypointense with a low signal intensity on T2-weighted MRI (5). Although ccRCC typically exhibits a slightly high signal intensity on T2-weighted imaging (T2WI) compared with renal parenchyma, 4%–21% of ccRCC cases present a low signal intensity influenced by histopathologic confounders such as a high nucleus-to-cytoplasm ratio and hemorrhage (6). Therefore, efficient methods, based on imaging parameters, are needed to establish an accurate differential diagnosis.

While both share heterogeneous textures and rounded shapes with partial renal infiltration (7), AML.wovf and ccRCC still present subtle differences. CT is a useful imaging tool to initially differentiate AML.wovf from ccRCC due to its sensitivity to fat, calcification, and cystic components, but is inferior in terms of soft tissue resolution, functional imaging, and safety (3). MRI, particularly with diffusion-weighted imaging (DWI) and phase-contrast imaging (8, 9), excels in diagnosing renal lesions, distinguishing false capsules rich in fibrous tissue, and highlighting lesion enhancement. Consequently, MRI could provide significant clues for differentiating between AML.wovf and ccRCC.

Previous studies have explored the potential of noninvasive MRI in differentiating between AML.wovf and ccRCC (10). Wataru et al. reported that the standard deviation of the apparent diffusion coefficient (ADC) combined with or without the T2 signal intensity (SI) ratio exhibits the highest performance in differentiating small AML.wovf from ccRCC (11). Park et al. revealed a significantly lower ADC in RCC than in AML.wovf (12). Instead of a single MRI sequence, a combination of multiple sequences is expected to increase the accuracy of differentiating between AML and ccRCC. Thus far, there has been a lack of a radiomics model constructed based on a structural T2WI and ADC in MRI that can differentiate between AML.wovf and ccRCC. In the present study, we constructed a nomogram to visualize the potential of a combination of MRI parameters in differentiating AML.wovf from ccRCC and validated its diagnostic accuracy.

## Materials and methods

### Participants

**Figure 1:**
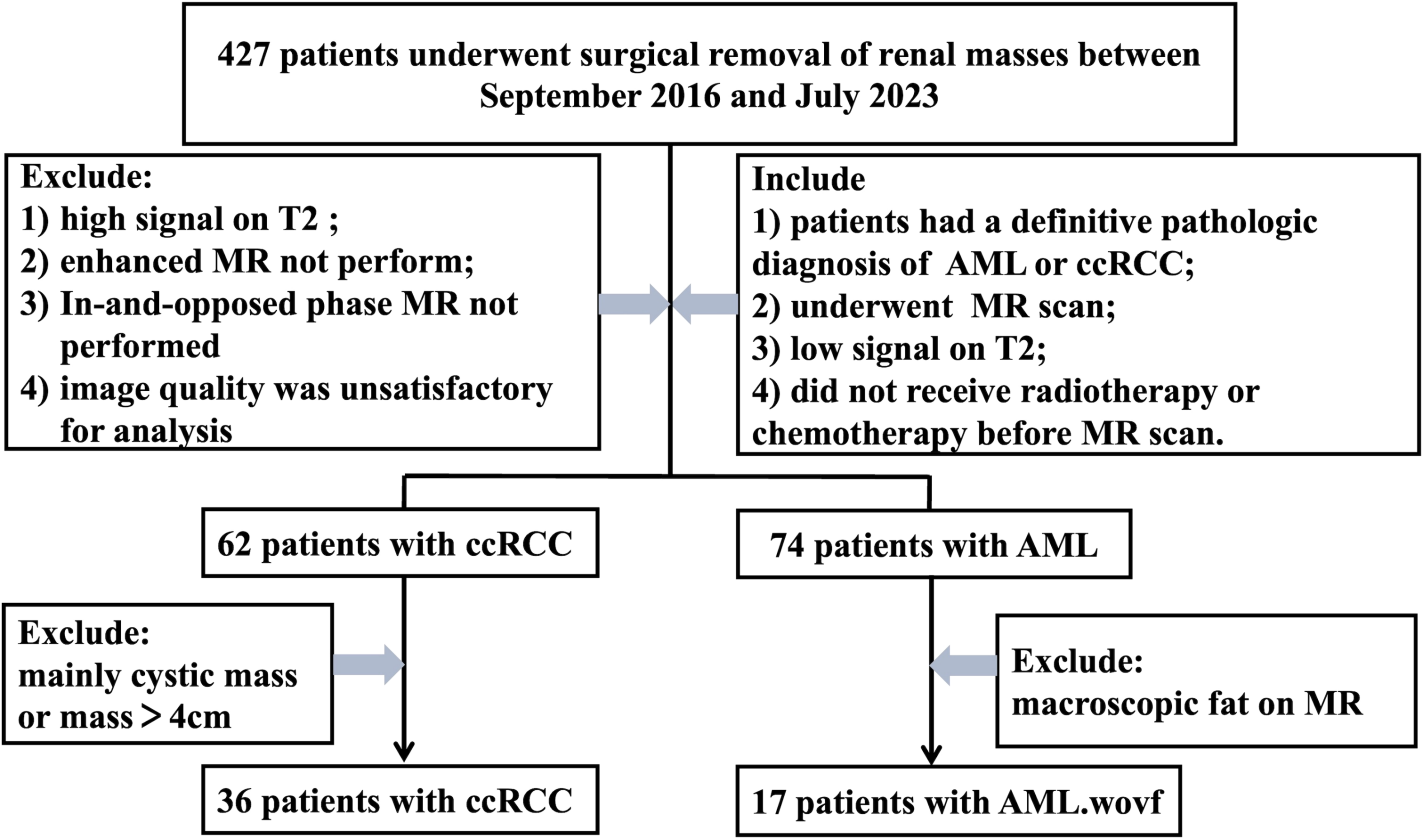
Flowchart of participant recruitment.

From September 2016 to July 2023, a total of 36 patients with ccRCC and 17 patients with AML.wovf confirmed by pathological analysis in the Affiliated Hospital of Nanjing University of Chinese Medicine were retrospectively recruited (**Figure 1**). All participants were examined using MRI, showing a low signal on T2WI. Those with a history of radiotherapy or chemotherapy, cystic mass or mass >4 cm with a diagnosis of ccRCC, and macroscopic fat on MRI with a definitive diagnosis of AML.wovf were excluded. The clinical data of the eligible participants were collected.

### MRI protocols

MRI was performed using the Siemens Magnetom Verio 3.0T MRI scanner (Siemens Healthcare, Erlangen, Germany) and an eight-channel body phased array coil. With the patients lying in a supine position on their back with the face up, MRI scans with the following sequences were captured: axial T1-weighted imaging (T1WI) [repetition time (TR) = 139 ms, echo time (TE) = 4.76 ms, slice thickness = 6 mm, interslice gap = 1 mm]; axial T2WI (TR = 1,900 ms, TE = 76 ms, slice thickness = 6 mm, interslice gap = 1 mm); sagittal fat-suppressed T2WI (T2WI-FS) phase (TR = 263 ms, TE = 4.76 ms, slice thickness = 5 mm, interslice gap = 1 mm); and DWI with a single-shot echo planar imaging sequence in sagittal and coronal planes (*b*-values of 50 and 800 s/mm^2^, respectively; TR = 6,900 ms, TE = 80 ms, slice thickness = 6 mm, interslice gap = 0).

### Imaging analysis

Two experienced gastrointestinal radiologists who were blind to the pathological outcomes were independently responsible for analyzing the MRI scans. MRI features were observed, including the location of the tumor (the left or right kidney), the pattern of growth (ball type or bean type), the edge (clear or unclear), the maximum diameter of the tumor, the texture of the tumor (homogenous or heterogeneous), special imaging signs (e.g., wedge-shaped sign, round tumor–kidney interface, pseudocapsule formation, and hemorrhage), the ADC, and the arterial-to-delayed enhancement ratio (ADER).

In particular, a wedge-shaped sign describes a renal mass with a triangular shape and with one point pointing toward the renal hilum. A round tumor–kidney interface indicates a circular interface between the tumor and the kidney. Pseudocapsule formation is defined as an unenhanced arc area located between the tumor and the renal parenchyma. ADER was calculated by dividing the difference in the signal intensity between the arterial and pre-contrast phases by the difference in the signal intensity between the delayed and pre-contrast phases (13). The ADC within the circular or oval regions of interest (ROIs) was calculated from a series of MRI scans acquired with different *b*-values. The ROIs for both ccRCC and AML.wovf lesions were positioned to encompass the maximum lesion areas while avoiding the most peripheral portions to exclude volume averaging.

### Histological examination

Surgically resected specimens were fixed in 10% formalin and immersed in hematoxylin and eosin (H&E) for H&E staining. In addition, immunohistochemical staining was conducted using antibodies against HBM-45, Melan-A, SMA, S-100, and Ki-67.

### Statistical analysis

Statistical analysis was performed using PASW Statistics 20.0 (IBM, SPSS, Armonk, NY, USA). The clinical and histopathological features of AML.wovf and ccRCC were compared using *t*-tests, the Mann–Whitney *U* test, and Fisher’s exact test, as appropriate. Univariate and multivariate logistic regression analyses were performed to identify predictors of ccRCC. Receiver operating characteristic (ROC) curves were plotted to determine the diagnostic performance, together with calculations of the optimal cutoff, Youden index, sensitivity, and specificity. Consistency in data interpretation was measured with the intraclass correlation coefficient (ICC). A significant difference was set at *p* < 0.05.

### Ethical considerations

The conduct of the study complied with the Declaration of Helsinki and was approved by the Institutional Review Board of The Affiliated Hospital of Nanjing University of Chinese Medicine (YJZ202119). Informed consent was waived.

## Results

### Clinical characteristics

Of the 17 patients with AML.wovf, eight were men and nine were women, with a mean age of 57.06 ± 9.82 years (range, 35–71 years). The cohort of 36 ccRCC patients consisted of 20 men and 16 women, with a mean age of 60.61 ± 9.98 years (range, 39–85 years). The diagnosis of AML.wovf or ccRCC was made by histological examination of biopsy samples. No significant differences were found in age, gender, and symptoms of lumbago and hematuria between the AML.wovf and ccRCC groups (all *p* > 0.05) (**Table 1**).

**Table 1:** Clinical features of AML.wovf *versus* ccRCC.

Characteristics	AML.wovf (*n* = 17)	ccRCC (*n* = 36)	*p*-value
Age (years)	57.06 ± 9.82	60.61 ± 9.98	0.267
Gender			
Men	8	20	0.563
Women	9	16	
Hematuria			
Yes	0	1	1.000
No	17	35	
Lumbago			
Yes	4	13	0.548
No	13	23	

AML.wovf, angiomyolipoma without visible fat; ccRCC, clear cell renal cell carcinoma.

### Imaging features

There were no significant differences in the proportions of round tumor–kidney interface, hemorrhage, tumor location, growth pattern, tumor edge, and maximum tumor size between groups (all *p* > 0.05) (**Table 2**).

**Table 2:** Imaging features of AML.wovf *versus* ccRCC.

Characteristics	AML.wovf (*n* = 17)	ccRCC (*n* = 36)	*p*-value
Wedge-shaped sign			
Yes	7	5	0.027
No	10	31	
Round tumor–kidney interface			
Yes	8	21	0.441
No	9	15	
Hemorrhage			
Yes	1	10	0.141
No	16	26	
Tumor texture			
Homogenous	6	2	0.016
Heterogeneous	11	34	
Pseudocapsule formation			
Yes	1	30	<0.001
No	16	6	
ADC	1.26 ± 0.46	1.65 ± 0.47	0.007
ADER			
≥1.5	8	4	0.01
<1.5	9	32	
Tumor location			
Left kidney	11	19	0.413
Right kidney	6	17	
Growth pattern			
Ball type	15	25	0.253
Bean type	2	11	
Tumor edge			
Clear	15	35	0.493
Unclear	2	1	
Maximum size (mm)	28.47 ± 27.76	20.97 ± 20.98	0.828

AML.wovf, angiomyolipoma without visible fat; ccRCC, clear cell renal cell carcinoma; ADER, arterial-to-delayed enhancement ratio; ADC, apparent diffusion coefficient.

The wedge-shaped sign, a homogenous tumor texture, and ADER ≥1.5 were more frequently observed on the MRI scans of patients with AML.wovf, while pseudocapsule formation was a typical sign in patients with ccRCC (all *p* < 0.05) (**Table 2**). The mean ADC was significantly lower in the AML.wovf group than in the ccRCC group (*p* < 0.05).

**Figure 2:**
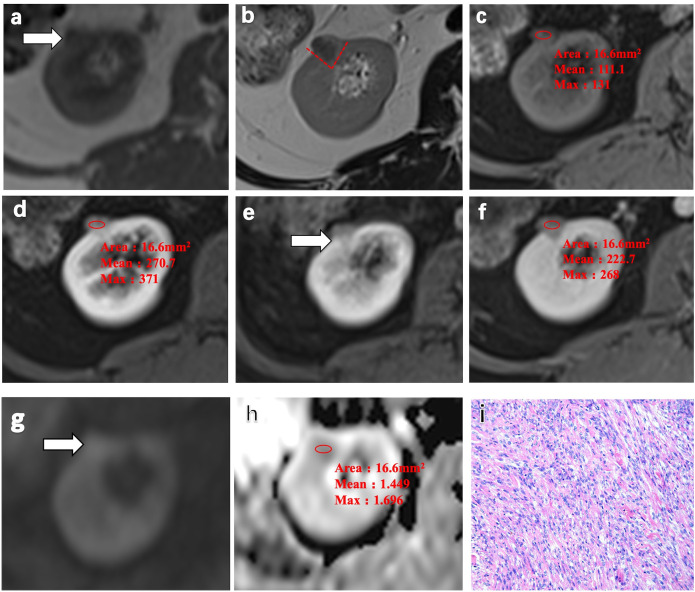
Representative MRI scans and pathology of a 64-year-old woman with angiomyolipoma without visible fat (AML.wovf) in the right kidney. **(a)** Homogeneous isointensity on T1-weighted imaging (T1WI). **(b)** Homogeneous hypointensity and the wedge-shaped sign (*red dashed line*) on T2-weighted imaging (T2WI). **(c)** Isointense lesion on the fat-suppressed, axial T1-weighted image. (**d**–**f**) Slight enhancement in the arterial phase of the lesion, which exited in the medullary phase and the delayed phase on the contrast-enhanced MRI scans. The arterial-to-delayed enhancement ratio (ADER) of the tumor is 1.43. **(g, h)** Apparent diffusion coefficient (ADC) of the tumor indicating homogeneous hypointensity. **(i)** Multiple epithelioid cells with lack of adipocytes visualized by H&E staining (magnification, ×200).

Representative MRI scans of a 64-year-old woman with AML.wovf in the right kidney consistently showed these imaging features, including homogeneous isointensity on T1WI and homogeneous hypointensity and a wedge-shaped sign on T2WI (**Figures 2a–c**). Contrast-enhanced MRI (CE-MRI) scans visualized a slight enhancement in the arterial phase of the lesion, which exited in the medullary and delayed phase, with an ADER of 1.43 (**Figures 2d–f**). The ADC of AML.wovf lesions indicated homogeneous hypointensity (**Figures 2g, h**). In addition, H&E staining of the AML.wovf lesions showed multiple epithelioid cells with lack of adipocytes (**Figure 2i**).

**Figure 3:**
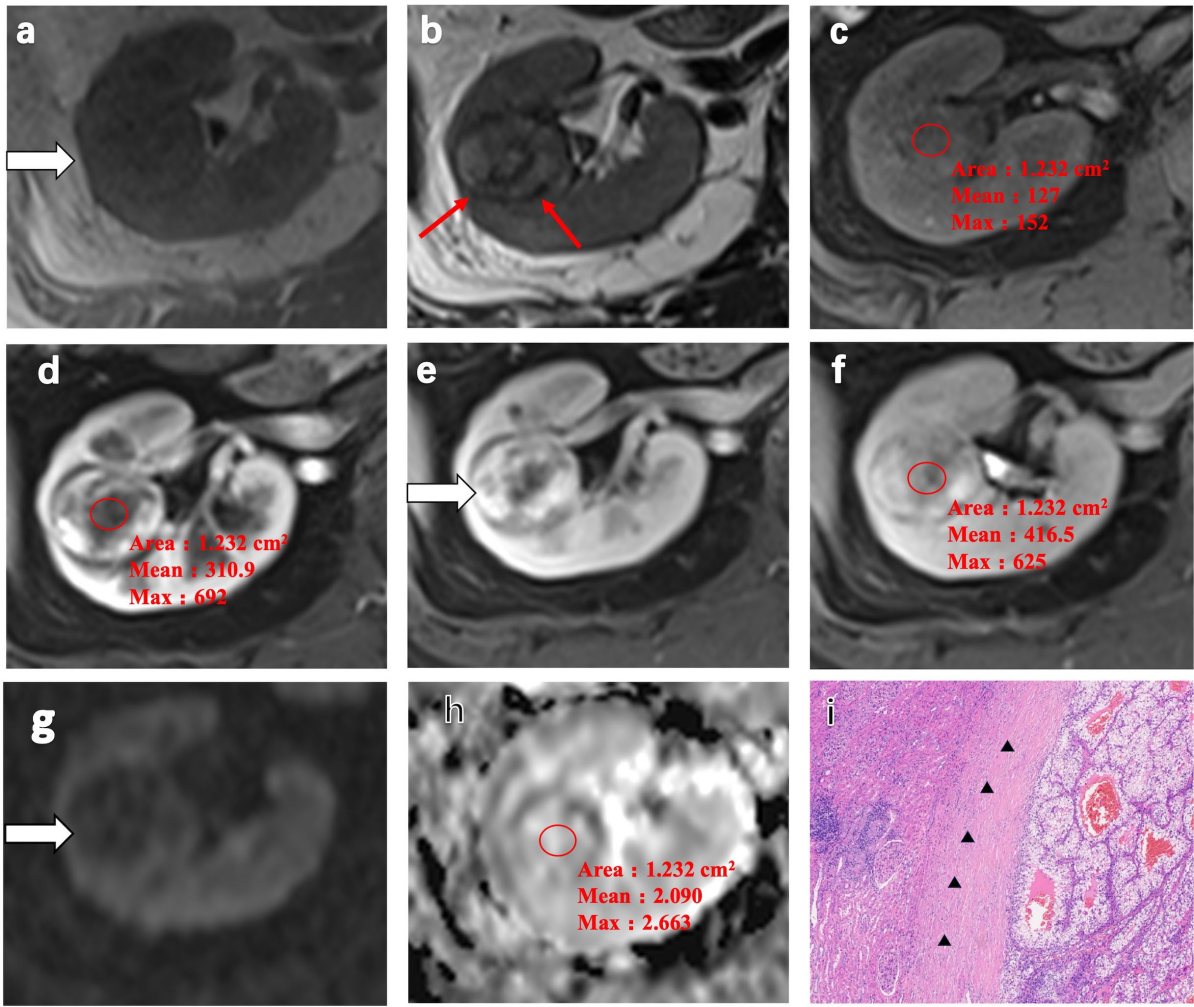
Representative MRI scans and pathology of a 52-year-old woman with clear cell renal cell carcinoma (ccRCC) in the right kidney. **(a)** Heterogeneous isointensity on T1-weighted imaging (T1WI). **(b)** Heterogeneous hypointensity and pseudocapsule formation (*red arrow*) on T2-weighted imaging (T2WI). **(c)** Isointense lesion on the fat-suppressed, axial T1-weighted image. **(d–f)** Heterogeneous avid arterial wash-in and quick wash-out in the medullary phase and the delayed phase on the contrast-enhanced MRI scans. The arterial-to-delayed enhancement ratio (ADER) of the tumor is 0.64. **(g, h)** Apparent diffusion coefficient (ADC) of the tumor indicating slight hyperintensity. **(i)** H&E staining showing expansive tumor growth that compressed the surrounding renal parenchyma, forming a fibrous pseudocapsule (*black arrow*) (magnification, ×100).

In a 52-year-old woman with ccRCC in the right kidney, homogeneous isointensity was observed on T1WI and heterogeneous hypointensity and pseudocapsule formation observed on T2WI (**Figures 3a–c**). CE-MRI scans visualized a heterogeneous avid arterial wash-in and a quick wash-out in the medullary and delayed phase, with an ADER of 0.64 (**Figures 3d–f**). The ADC of ccRCC indicated slight hyperintensity (**Figures 3g, h**). Expansive tumor growth compressed the surrounding renal parenchyma, forming a fibrous pseudocapsule in H&E staining (**Figure 3i**).

### Predictors to differentiate AML.wovf from ccRCC

Multivariate logistic regression analysis showed that the wedge-shaped sign (OR = 0.05, 95%CI = 0.00–0.97, *p* = 0.047), pseudocapsule formation (OR = 140.29, 95%CI = 4.92–3996.35, *p* = 0.004), and the ADC (OR = 36.22, 95%CI = 1.23–1064.38, *p* = 0.037) were independent predictors that differentiated AML.wovf from ccRCC (**Table 3**).

**Table 3:** Predictive factors of ccRCC identified by multivariate logistic regression.

Variable	*β*	SE	*Z*	*p*	OR (95%CI)
Intercept	−1.31	1.40	−0.93	0.350	0.27 (0.02–4.22)
Wedge-shaped sign					
No					1.00 (Reference)
Yes	−3.05	1.54	−1.98	**0.047**	0.05 (0.00–0.97)
Tumor texture					
Heterogeneous					1.00 (Reference)
Homogenous	−4.77	2.43	−1.96	0.050	0.01 (0.00–0.99)
Pseudocapsule formation					
No					1.00 (Reference)
Yes	4.94	1.71	2.89	**0.004**	140.29 (4.92–3,996.35)
ADC custom					
1					1.00 (Reference)
2	3.59	1.72	2.08	**0.037**	36.22 (1.23–1,064.38)
ADER custom					
1					1.00 (Reference)
2	−3.33	1.85	−1.80	0.072	0.04 (0.00–1.35)

ccRCC, clear cell renal cell carcinoma; OR, odds ratio; CI, confidence interval; ADC, apparent diffusion coefficient; ADER, arterial-to-delayed enhancement ratio

The meaning of the bold values: p<0.05.

**Figure 4:**
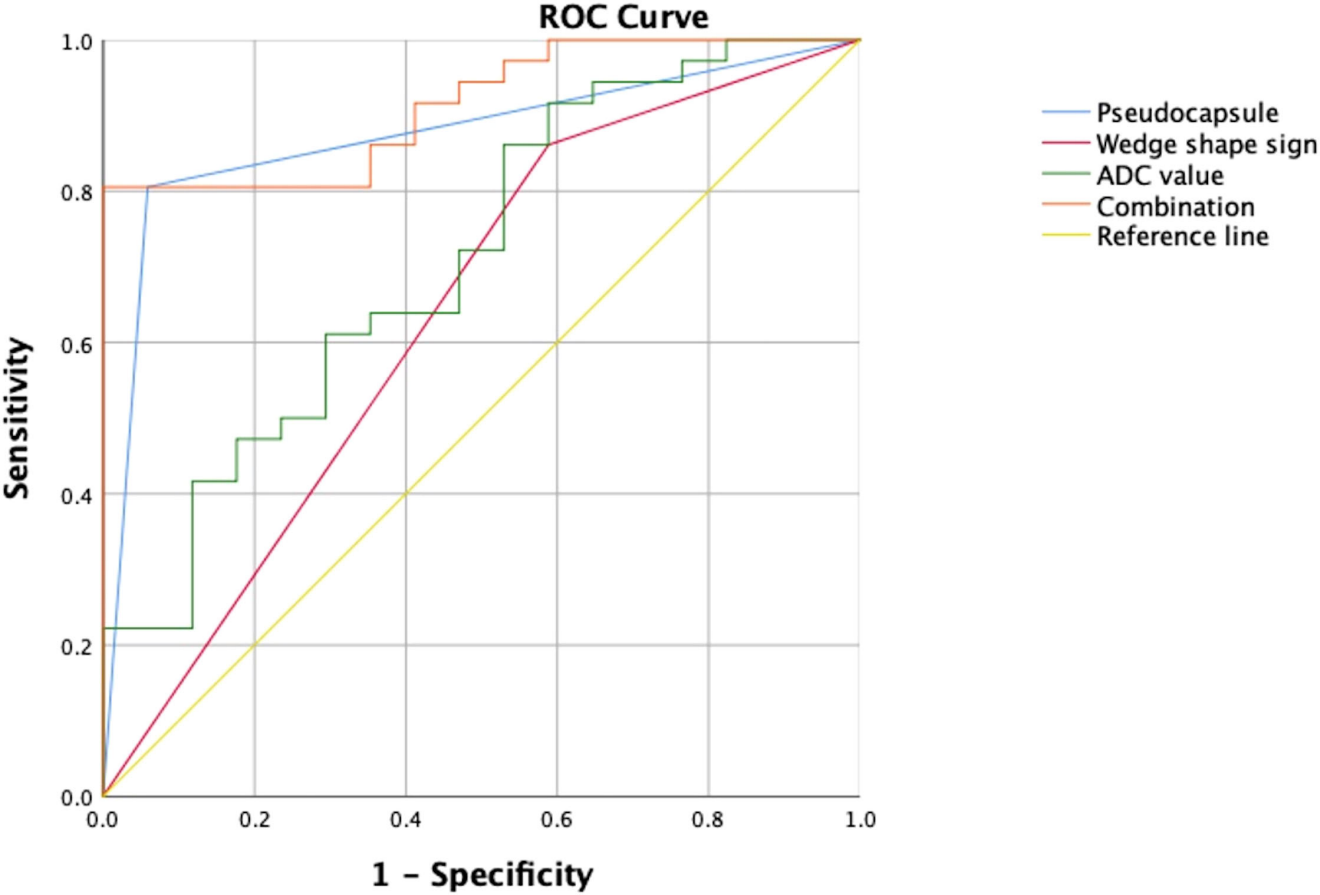
Performance of the risk factors in differentiating angiomyolipoma without visible fat (AML.wovf) from clear cell renal cell carcinoma (ccRCC) determined by receiver operating characteristic (ROC) analysis. The areas under the curve (AUC) of the wedge-shaped sign, pseudocapsule formation, and apparent diffusion coefficient (ADC) were 0.636 (95%CI = 0.467–0.805), 0.873 (95%CI = 0.771–0.976), and 0.711 (95%CI = 0.561–0.861), respectively. The AUC of their combination was 0.913 (95% CI = 0.84–0.987).

The area under the curve (AUC) of the combination of the wedge-shaped sign, pseudocapsule formation, and ADC was 0.913 (95%CI = 0.840–0.987), which was much larger than those of the single predictors (AUC = 0.636, 95%CI = 0.467–0.805; AUC = 0.873, 95%CI = 0.771–0.976; and AUC = 0.711, 95%CI = 0.561–0.861, respectively) (**Table 4**, **Figure 4**).

**Table 4:** Performance of each predictive factor and their combination in differentiating AML.wovf from ccRCC.

	AUC	95%CI	Sensitivity (%)	Specificity (%)
Wedge-shaped sign	0.636	0.467–0.805	86.1	41.2
Pseudocapsule formation	0.873	0.771–0.976	80.6	94.1
ADC	0.711	0.561–0.861	86.1	47.1
Combination	0.913	0.840–0.987	80.6	100

AML.wovf, angiomyolipoma without visible fat; ccRCC, clear cell renal cell carcinoma; AUC, area under the curve; CI, confidence interval; ADC, apparent diffusion coefficient

### Construction of a nomogram to differentiate AML.wovf from ccRCC

**Figure 5:**
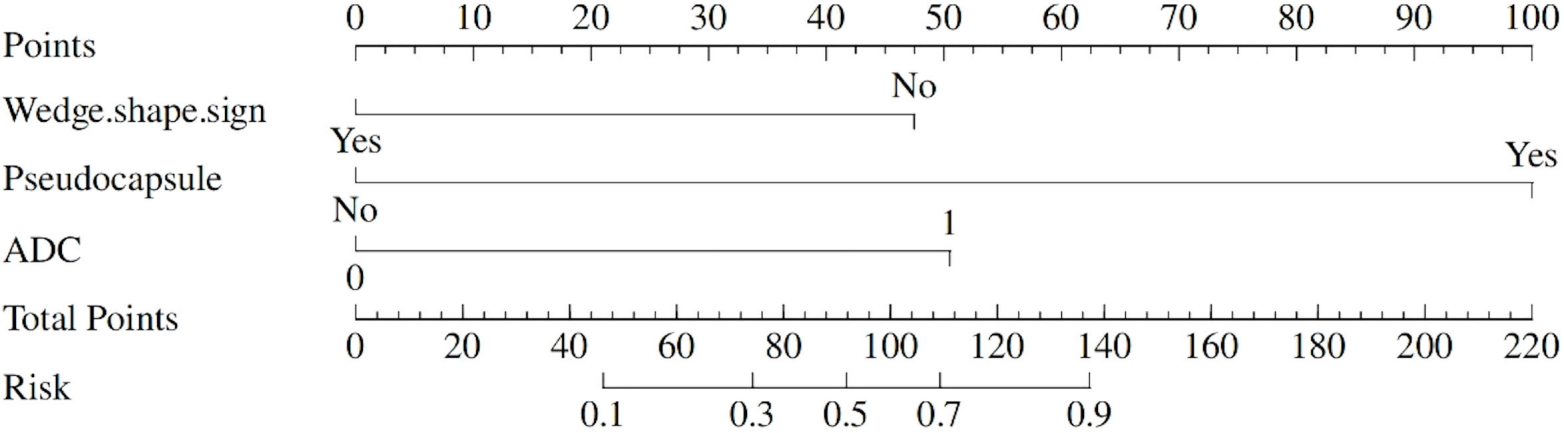
A nomogram to predict the likelihood of developing clear cell renal cell carcinoma (ccRCC).

To facilitate clinical application, an optimal cutoff value of 1.161 points was established through ROC curve analysis. A nomogram incorporating wedge-shaped sign, pseudocapsule formation, and ADC was created to visualize their potential in differentiating between AML.wovf and ccRCC (**Figure 5**). By adding up the scores (bottom scale) corresponding to each predictive indicator on the top scale of the chart, a total score was calculated to quantify the risk of ccRCC.

## Discussion

MRI can assist in differentiating between AML.wovf and ccRCC and in avoiding unnecessary surgeries. Prior studies have highlighted the diagnostic potentials of MRI-based radiomics models and ADC (11, 12, 14). However, previous studies have mainly explored the clinical significance of CT or ultrasound in renal tumor diagnostics, with a lighter emphasis on MRI, particularly ADC and ADER (15, 16). In this study, characteristic MRI features that differentiate AML.wovf from ccRCC were identified.

On T2WI, ccRCC typically appears as hyperintense. However, previous studies have indicated that the hypointense signals on T2WI vary between 4% and 21% among the total population of ccRCC cases (12, 17). ccRCC presents a low T2WI signal due to the proliferation of fibrous tissue that restricts water molecule movement and shortens the T2 relaxation time. Meanwhile, the T2 signal intensity is further reduced by the paramagnetic properties of hemosiderin deposition from hemorrhage and local magnetic fields disrupted by tumor calcification.

The renal cortex is gradually compressed during the growth of renal tumors, creating a flat interface with the renal parenchyma and thus forming a wedge-shaped sign. Generally, this feature indicates benign tumors such as AML and reflects a low ability of infiltration and growth along paths of least resistance, such as interlobular spaces. In the present study, the incidence of a wedge-shaped sign was significantly higher in the AML.wovf group than in the ccRCC group, consistently supporting the benign property of AML.wovf (18). Other imaging characteristics of a benign tumor, such as a homogenous tumor texture and ADER ≥1.5, were also frequently observed in the AML.wovf group.

The characteristic hypointense rim, known as the pseudocapsule that delineates renal tumors from adjacent tissues, was initially documented in MRI studies by Hricak et al. in 1985 (19). This distinctive feature presents as a low-signal boundary encircling the neoplasm, positioned between the lesion and either the normal renal tissue or the perirenal adipose tissue, and is observable across both T1- and T2-weighted sequences. Histological correlation suggests that this signal pattern corresponds to the composition of fibrous tissue (20). Among various MRI sequences, T2WI has demonstrated superior sensitivity in visualizing the pseudocapsule, particularly due to the enhanced contrast between the hyperintense tumor and the relatively lower signal intensity of the surrounding renal parenchyma (21). Pseudocapsule formation often indicates malignancy (22). Consisting of a fibrous layer and a compressed renal tissue, a pseudocapsule appears as an unenhanced arc on images. Previous data showed the presence of a pseudocapsule in 66%–90% of small RCCs, but only in 0%–10% of AML.wovf cases (23, 24). In this study, the formation of pseudocapsule was observed in the majority of ccRCC cases, and its incidence was significantly higher than that in the AML.wovf group (83.3% *vs*. 5.9%).

DWI is a technique that reveals the diffusion (thermal motion or the Brownian motion) of water molecules in biological tissues. The ADC measurement reflects the random thermal motion of protons and quantifies the level of diffusion (25). The noninvasive evaluation of ADC on DWI scans greatly favors an imaging-based diagnosis of small tumors with a low T2WI signal. In contrast to the findings of Park et al. (12), our results indicated that the ADC was significantly lower in the AML.wovf group compared with the ccRCC group. This discrepancy may be attributed to the fact that our study focused exclusively on ccRCC, whereas Park et al. included other renal cancer subtypes, such as papillary RCC and chromophobe RCC, in their analysis. Li et al. demonstrated that the ADC values of non-ccRCC were lower than those of ccRCC, which is consistent with our findings (26). Tanaka et al. consistently reported a lower mean (0.80 × 10^−3^ *vs*. 1.54 × 10^−3^ mm^2^/s) and maximum ADC values (0.93 × 10^−3^ *vs*. 2.15 × 10^−3^ mm^2^/s) in AML.wovf compared with ccRCC, which could probably be attributed to the restricted water diffusion caused by smooth muscle cells and adipose tissue (27).

The implementation of a 1.161-point cutoff provides clinicians with a clear boundary for decision-making. Patients with scores above this threshold may require more intensive evaluation or intervention, while those with lower scores might avoid unnecessary invasive procedures.

This study has a number of limitations. Firstly, this is a single-center study with a small sample size that excluded cases of AML.wovf and ccRCC with high T2 signal intensity. Secondly, the retrospective study design was prone to recall, selection, and observer biases.

## Conclusion

The combination of MRI parameters with different sequences, including the wedge-shaped sign, pseudocapsule formation, and the ADC, demonstrated high diagnostic accuracy in differentiating between AML.wovf and ccRCC.

## Data Availability

The original contributions presented in the study are included in the article/supplementary material. Further inquiries can be directed to the corresponding authors.
